# The underappreciated role of rifampin in shaping the epidemiology of RT027-like *Clostridioides difficile* lineages

**DOI:** 10.1093/jac/dkag310

**Published:** 2026-09-04

**Authors:** Isabella A Tickler, Richard V Goering, Fred C Tenover

**Affiliations:** Department of Medical and Scientific Affairs, Cepheid, 904 Caribbean Drive, Sunnyvale, CA 94089, USA; Department of Medical Microbiology and Immunology, Creighton University, Omaha, NE 68178, USA; College of Arts and Sciences, Department of Biology, University of Dayton, Dayton, OH 45469, USA

## Abstract

The global emergence and dissemination of epidemic *Clostridioides difficile* ribotype 027 (RT027) and related lineages illustrate the role of antimicrobial pressure in shaping the epidemiology of this pathogen. We propose a hierarchical model in which fluoroquinolones (FQs) served as the dominant selective force underlying the worldwide expansion of RT027-related strains, with rifampin and rifamycins acting as secondary modifiers that promoted persistence and regional dominance.

The emergence and global dissemination of epidemic *C. difficile* ribotype 027 (RT027) and the closely related clade 2 lineages provide a clear example of how antimicrobial pressure can shape a pathogen’s evolution and epidemiology.^1^ Although FQs were the dominant selective force driving the transition of RT027 from a non-epidemic strain in the 1980s to a leading cause of outbreaks across two continents in the early 2000s, accumulating evidence points to a contributory role for rifampin and rifamycins in sustaining and entrenching RT027-like strains in specific regions.^2,3^

The temporal overlap between the widespread clinical adoption and usage of FQs, particularly later-generation agents, such as ciprofloxacin and moxifloxacin, and the explosive rise of *C. difficile* RT027 strongly supports a causal relationship (Figure 1).^4^ RT027 and related clade 2 strains harbour characteristic mutations in *gyrA* and *gyrB* that confer high-level FQ resistance to the organism without an apparent fitness cost. Resistance to FQs provided a marked selective advantage in healthcare settings with high FQ use, allowing RT027 to expand clonally and dominate outbreaks across North America and Europe in the early 2000s.^4^ The subsequent decline of RT027 in multiple countries following reductions in FQ use and implementation of antimicrobial stewardship interventions further supports the central role of FQs in the emergence and expansion of epidemic RT027.^4^

**Figure 1.**
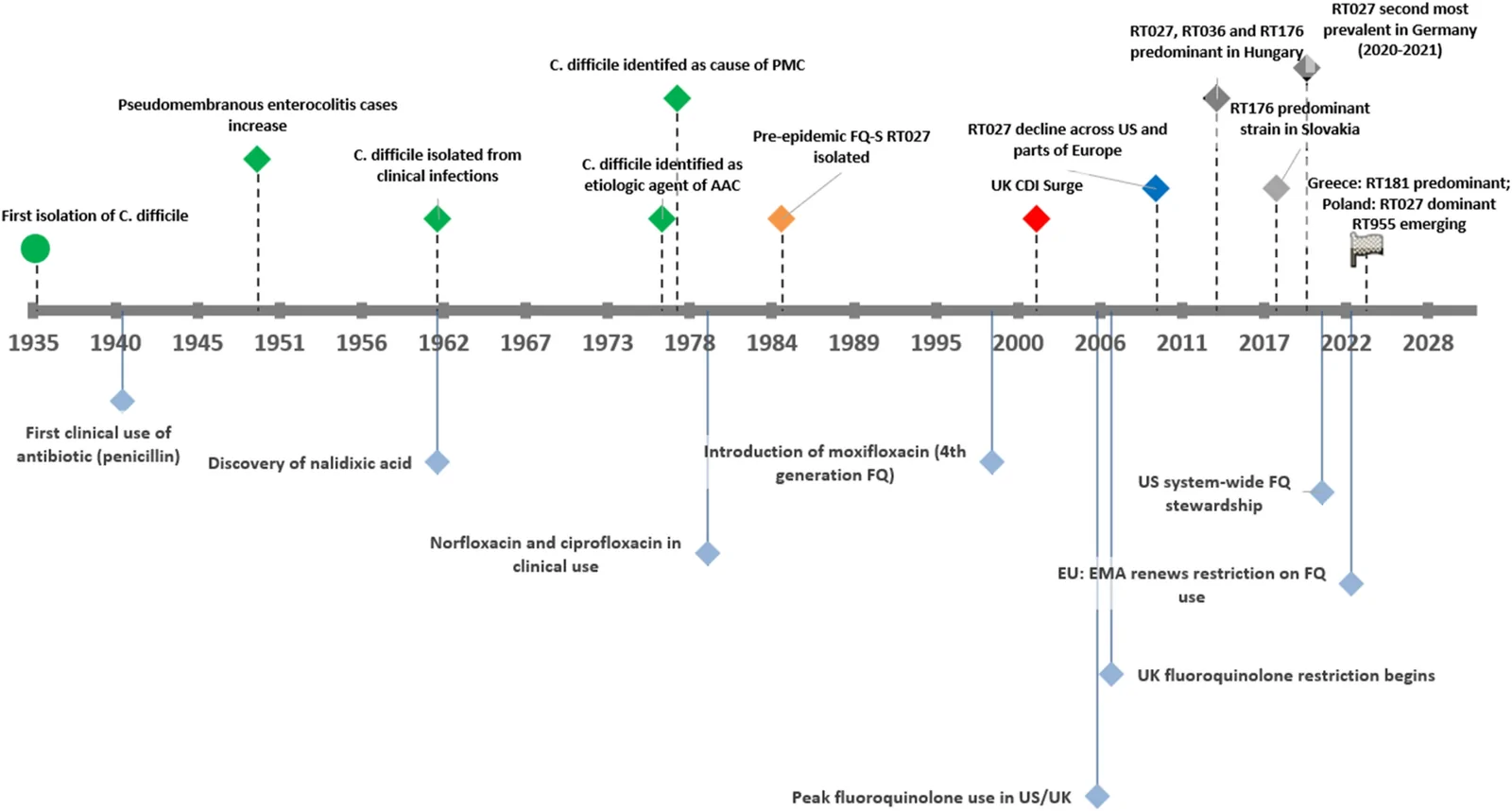
FQ milestones (shown in lower timeline in blue) versus *C. difficile* epidemiological shifts (upper timeline). Main events pertaining to isolation and identification of *C. difficile* as a pathogen are labelled in green. Pre- and post-epidemic milestones are indicated in orange and red, respectively. The beginning of the decline of RT027 is marked in blue. Main differentiating events of RT027-like strains are labelled in grey.

As shown in Figure 1, multiple national experiences further reinforced the link between RT027 and FQs. In the UK and parts of the USA, sharp declines in RT027 prevalence closely followed FQ restriction policies, whereas countries with sustained or higher FQ consumption continued to report circulation of RT027-like lineages (e.g. RT176, RT181, RT955).^4–7^ Importantly, this effect appears across the class of FQ agents: stewardship interventions targeting FQs as a group, rather than substitution within the class, were the most effective in reducing the prevalence of RT027 and RT027-like strains in several regions.^8,9^ Together, these data establish FQs as the dominant evolutionary driver underlying the global expansion of these FQ-resistant RT027 strains, and subsequent decline and diversification, of RT027 once the selective pressures were removed. These epidemiologic data, however, paint a picture of antimicrobial pressure as monolithic, while we argue that it is much more layered and complex.

Although less central than FQs, rifamycin exposure appears to have exerted additional selective pressure on RT027 and related strains. For clarity, we will use ‘rifampin’ when referring to susceptibility data and *rpoB* mutations and ‘rifamycins’, such as rifampin, rifabutin, rifaximin and rifapentine, when referring to class-level prescribing pressure. Rifampin resistance in *C. difficile* arises through mutations in the *rpoB* gene, several of which (e.g. R505K and H502N) are repeatedly observed in RT027, the RT027-related ribotypes, RT176 and RT181, and other emerging RT027-like ribotypes, such as RT955 (Figure 2).^2,6,10,11^ These mutations are associated with intermediate to high-level rifampin minimal inhibitory concentrations (MICs) and have been linked to documented outbreaks of rifampin-resistant RT027 strains.^12^ Furthermore, resistance to rifamycins can emerge rapidly during therapy through acquisition of *rpoB* mutations, highlighting the potential for localized selection under sustained drug exposure.^13^

**Figure 2.**
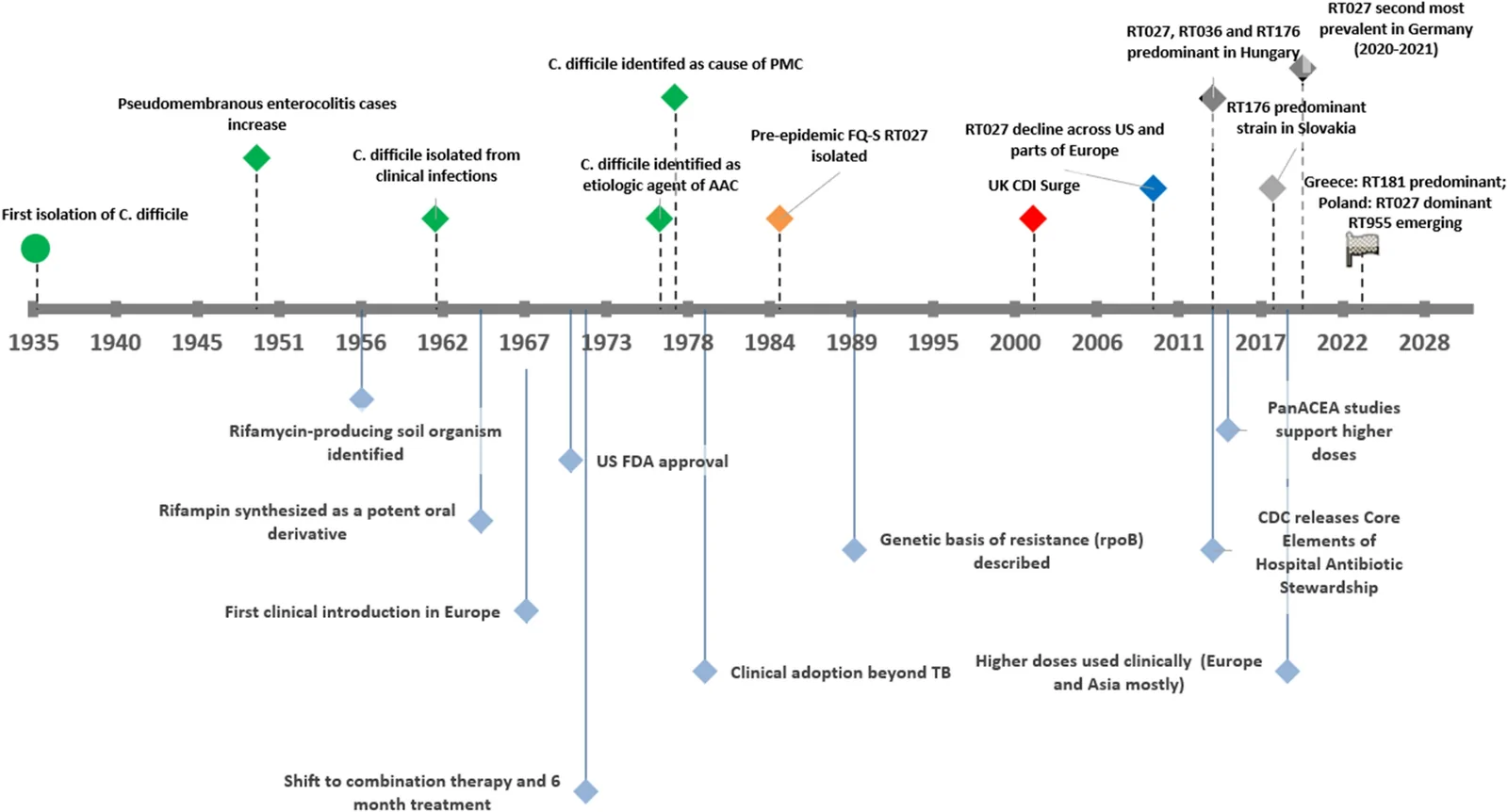
Rifampin milestones (lower portion of the timeline) and *C. difficile* epidemiological shifts (upper timeline). Main events pertaining to isolation and identification of *C. difficile* as a pathogen are labelled in green. Pre- and post-epidemic milestones are indicated in orange and red, respectively. The beginning of the decline of RT027 is marked in blue. Main differentiating events of RT027-like strains are labelled in grey.

Unlike FQs, rifampin and other rifamycins are not broadly prescribed across hospital populations, but their intensive use in specific clinical contexts, most notably tuberculosis, orthopaedic and prosthetic joint infections, gastrointestinal infections, and hepatic encephalopathy, creates localized but strong selection pressures supporting the emergence of rifamycin-resistant isolates of *C. difficile.*^3,12,14–16^ This hypothesis is supported by observations from Germany, where a cluster of CDI cases among patients with osteoarticular infections included numerous rifampin-resistant RT027 isolates in a population receiving antimicrobials commonly used for orthopaedic infections.^17^ Similarly, a highly rifampin-resistant RT046 outbreak was reported among tuberculosis patients in Poland, providing another example of how concentrated rifampin exposure may select for resistant *C. difficile* lineages even outside the RT027 complex.^18^ In South Africa, RT017 strains isolated from patients attending tuberculosis hospitals were frequently co-resistant to rifampin and FQs, suggesting that anti-tuberculosis treatment practices may influence regional *C. difficile* population structures.^19^ Recent studies, including those from the PanACEA programme (Pan-African Consortium for the Evaluation of Antituberculosis Antibiotics; https://panacea-tb.net/clinical-studies/panacea-studies/), have focused on higher-dose rifampin strategies to shorten TB treatment. However, the geographic settings in which such dose optimization approaches have been evaluated do not overlap with regions where RT027-like *C. difficile* lineages have emerged or become predominant.^20^

A 2011 study demonstrated that rifamycin resistance in *C. difficile* may be associated with national rifamycin use, with markedly higher resistance rates in Italy compared with Canada, i.e. countries with high versus low rifamycin consumption, respectively.^16^ The absence of rifamycin resistance among the RT027 strains from Canada further supported the important role of antimicrobial exposure.^16^ Similarly, epidemiologic observations from Germany, Poland and Greece suggest that rifampin resistance among *C. difficile* isolates may have contributed to the persistence and regional dominance of RT027-like lineages once they were established, rather than serving as the initial driver of their emergence.^10,21,22^ Notably, RT181, an 027-related lineage now predominant in parts of Greece and Romania, exhibits high rifampin MICs, raising the possibility that rifampin exposure helped shape its ecological niche in that region.^10^ Although the burden of *Mycobacterium tuberculosis* (TB) infection in Greece is comparatively low, widespread antimicrobial prescribing practices, including the use of rifampin for non-TB indications, may be contributing to the selective evolutionary pressure that maintains RT181 strains in that population.^23^ RT955, an 027-like strain type reported to cause outbreaks in the UK and Poland, is characterized by both rifampin and metronidazole resistance and also has been circulating in Serbia since 2018.^22^ However, the Serbian RT955 lineage lacks the genetic determinants associated with rifampin and metronidazole resistance.^22^ The Serbian RT955 lineage likely reflects independent adaptation to regional antimicrobial use patterns or represents a distinct lineage within the same ribotype.^22^ The underlying drivers that are sustaining the presence of the RT955 lineage in Serbia are likely multifactorial.

Taken together, the data support a hierarchical impact of antimicrobial selection among *C. difficile* RT027 and RT027-like strains. FQs acted as the primary trigger, enabling the initial expansion and global spread of RT027 and related clade 2 lineages by strongly favouring FQ-resistant genotypes. Unlike FQ resistance, rifamycin resistance appeared to drive the local emergence and dissemination of RT027-like strains, like RT955, rather than exerting global pressure that would drive more far-reaching emergence and stabilization of the strains. Use of rifamycins, which was much more variable from region to region, likely resulted in more localized ecological fitness within those areas of high usage. Thus, rifamycins appeared to function as a secondary modifier after FQ use, potentially reinforcing the success of already established clade 2 strains in environments where rifamycins use was common or prolonged. Additional antibiotic classes (e.g. cephalosporins, clindamycin) further amplify transmission risk by inducing gut dysbiosis, even if they do not select as directly for RT027.

Importantly, persistence of epidemic *C. difficile* lineages is likely multifactorial. Although antimicrobial resistance can confer a selective advantage, transmission dynamics, infection prevention practices, host susceptibility and strain-specific traits also influence regional success. The long-term success of RT018 in Italy illustrates this complexity, with rifampin resistance, enhanced adhesion, robust toxin production and increased sporulation all likely contributing to its predominance.^24^ Similar factors may contribute to the persistence of other RT027-like lineages.

The evolutionary trajectories of RT027 and RT027-like *C. difficile* strains suggest that epidemic success of clones and lineages is rarely driven by a single antimicrobial agent in isolation. Instead, layered selection pressures, dominated in this case by FQs and reinforced by agents such as rifampin, appear to shape both the emergence and regional persistence of high-risk lineages. This perspective reinforces the central role of sustained, class-level FQ stewardship, while highlighting rifamycin use as a potentially underappreciated contributor to RT027-like epidemiology. Integrating whole-genome sequencing, high-resolution core-genome phylogenetics, longitudinal genomic surveillance and antimicrobial consumption data will be critical to determining the extent to which rifamycin exposure contributes to the persistence and diversification of RT027 and related lineages. Such studies are critical to understanding the mechanisms underlying their continued success and informing future prevention and control strategies.
